# Effects of Botulinum Toxin Type A on Microvessels in Hypertrophic Scar Models on Rabbit Ears

**DOI:** 10.1155/2020/2170750

**Published:** 2020-06-16

**Authors:** Na Zhou, Dongping Li, Yanzhu Luo, Junping Li, Yuhong Wang

**Affiliations:** ^1^Aier School of Ophthalmology, Central South University, Changsha, China; ^2^Department of Oculoplastic Surgery, Hankou Aier Eye Hospital, Wuhan, China

## Abstract

**Background:**

Although Botulinum Toxin Type A (BTXA) has been applied to scar prevention and treatment, the mechanisms still require further exploration.

**Objective:**

To investigate the effects of BTXA on microvessels in the hypertrophic scar models on rabbit ears.

**Methods:**

Eight big-eared New Zealand rabbits (males or females) were selected to establish scar models. One ear of each rabbit (4 models in each ear) was selected randomly to be injected with BTXA immediately after modeling and included in the treated group, while the opposite ear was untreated and included in the control group. The growth of scars in each group was observed and recorded, and 4 rabbits were sacrificed on days 30 and 45 after modeling. Then, scar height was measured by hematoxylin-eosin (HE) staining, vascular endothelial growth factor (VEGF) expression was detected by immunohistochemical (IHC) testing, and microvessel density (MVD) was calculated based on CD34 (human hematopoietic progenitor cell antigen).

**Results:**

The wounds in each group were well healed and free from infection or necrosis. On days 30 and 45, the scar height, MVD value, and VEGF expression in the treated group were lower than those in the control group (*P* < 0.05). For the treated group, the above indicators on day 45 were lower than on day 30 (*P* > 0.05). Besides, there was a positive correlation between the MVD value and the VEGF expression in the treated group (*P* < 0.05).

**Conclusion:**

The injection of BTXA immediately after modeling inhibits VEGF expression and reduces angiogenesis, thereby inhibiting hypertrophic scar formation.

## 1. Introduction

As a pathological scar generally caused by burns, injuries, and surgery, the hypertrophic scar (HS) can affect the life quality of patients both physically and psychologically, for which reason it has long been a great challenge for clinicians. In recent years, BTXA has been successively reported to be effective in scar prevention and treatment [1–6], but the studies on related mechanisms mainly focus on BTXA inhibiting fibroblast generation, promoting apoptosis, and reducing collagen deposition [7, 8], and there were few reports on its effects on microvessels. However, as studies have shown that the hyperplasia of scar tissue is closely related to MVD and microcirculatory blood flow [9], could BTXA be effective in scar prevention and treatment by inhibiting angiogenesis? This study was performed to determine the effects of BTXA on blood vessels in HS by detecting the VEGF expression and MVD in the HS on rabbit ears injected with BTXA.

## 2. Materials and Methods

### 2.1. Subjects

Eight big-eared New Zealand rabbits (males or females) in healthy condition with sound ears, weighing 2–3 kg, were selected as subjects from the Laboratory Animal Center of Tongji Medical College, Huazhong University of Science and Technology. They were fed regularly in separate cages at an indoor temperature of 21–25°C with clean air ventilation in accordance with the *Policy on Humane Care and Use of Laboratory Animals*.

### 2.2. Reagents

The reagents are as follows: hematoxylin (Sigma), eosin Y (water-soluble), absolute ethanol, xylene, neutral balsam (Sinopharm Chemical Reagent Co., Ltd.), PBS solution (0.01 M), hematoxylin stain solution, H_2_O_2_, EDTA (pH 8.0) antigen retrieval solution (Wuhan Hundred Thousand Degree Biological Technology Co., Ltd.), BSA (bovine serum albumin) (BIOFROXX), DAB substrate kit (DAKO), VEGF primary antibodies (Proteintech Group), CD34 primary antibodies (BioWorld), and IHC secondary antibody kit (SeraCare).

### 2.3. Experimental Protocol

#### 2.3.1. Modeling

The rabbits were raised for one week before the study for acclimatization, fasted and forbidden to drink for 8 h before modeling, and then anesthetized with 3% pentobarbital sodium (30 mg/kg). According to the method proposed by Morris and Wu [10], the ventral side of a rabbit ear was disinfected with complex iodine 3 times; the full-thickness skin and perichondrium were drilled through with a 6 mm diameter ophthalmic corneal trephine; with the skin and perichondrium fully removed and the cartilage preserved, the wound was first pressed to stop the bleeding, then exposed after cleaning with normal saline. A total of 4 identical wounds were prepared on both sides along the long axis on each rabbit ear at an interval of 1.5–2 cm.

#### 2.3.2. Grouping

One ear of each rabbit was randomly selected to be injected with BTXA and included in the treated group, while the opposite ear was untreated and included in the control group. The growth of scars in each group was observed and recorded every 2 days, and 4 rabbits were sacrificed on days 30 and 45 after modeling.

#### 2.3.3. Method

*(1) Injection and Sampling*. BTXA (Lanzhou Biochemical Company, Lanzhou City, China), diluted with normal saline to 40 IU·mL^−1^, was subcutaneously injected with 30G needles at 3 and 9 o'clock on each wound approx. 3 mm under the skin at a dose of 2 U/point. The general condition of the wounds was observed every 1–2 days, and sampling covered scar tissue and surrounding normal tissues within a range of at least 2 mm.

*(2) Histological Examination*. Tissue specimens were fixed in 4% paraformaldehyde, dehydrated in ethanol at different gradients until transparent, and then embedded in paraffin, sliced into serial sections at 5 *μ*m thickness, and finally processed for HE staining and IHC staining.

*Scar height*: this refers to the highest vertical height between the epidermis at the top of the bulging scar tissue and cartilage, which was measured by a light microscope after HE staining.

*MVD calculation*: in the 3 views randomly selected from each section, the presence of brownish-yellow granules in the cytoplasms of VECs was considered as CD34-positive. Referring to the sampling and counting method of Weidner [11], the number of positive-stained vessels was counted under a 200× light microscope, and the mean value of the 3 views was counted as the MVD value (/scope).

*VEGF expression detection*: photos with identical brownish-yellow color were selected by Image-Pro Plus 6.0 (Media Cybernetics, Inc., Rockville, MD, USA) as the uniform criterion for judging the positivity of all photos, thus analyzing the integrated optical density (IOD) of the positivity of each photo.

### 2.4. Statistical Analysis

The measurement data was expressed as mean ± standard deviation (*X* ± *s*) and processed for statistical analysis using SPSS 18.0 statistical software. The Shapiro-Wilk test was applied to determine normality, and Levene's test was applied to assess homogeneity of variance. The scar height, MVD value, and VEGF expression were compared between the treated group and the control group using the *t*-test of two independent samples, the correlation between the two variables was determined using Pearson linear correlation analysis, and *P* < 0.05 indicated statistically significant difference.

## 3. Results

### 3.1. Gross Observation

**Figure 1:**
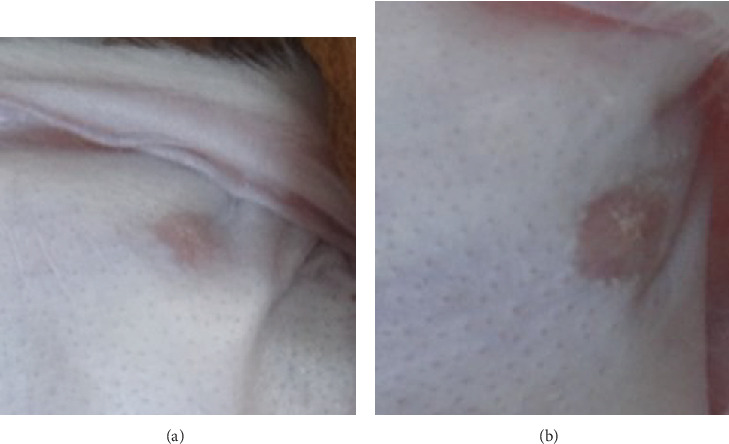
Gross appearance. (a) Gross appearance of treated group at day 45. (b) Gross appearance of control group at day 45.

The wounds in each group were completely healed in about 15–19 days and free from infection or necrosis. On days 30 and 45, the scars in the treated group were flatter than those in the control group and soft to the touch (Figure 1).

### 3.2. Scar Height

**Figure 2:**
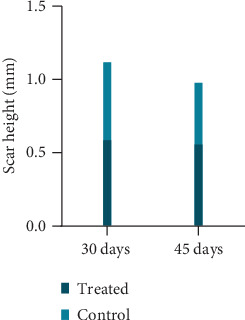
Scar height of two groups at each time point.

On days 30 and 45, the scar height of the treated group was lower than that of the control group, and the difference was statistically significant (*P* < 0.05); the scar height of the treated group on day 45 was lower than that on day 30 (*t* = 1.220, *P* = 0.232) (Table 1, Figure 2).

### 3.3. MVD

**Figure 3:**
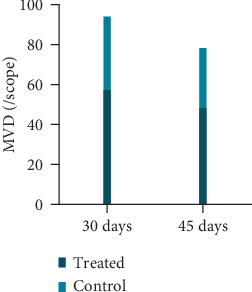
MVD value of two groups at each time point.

**Figure 4:**
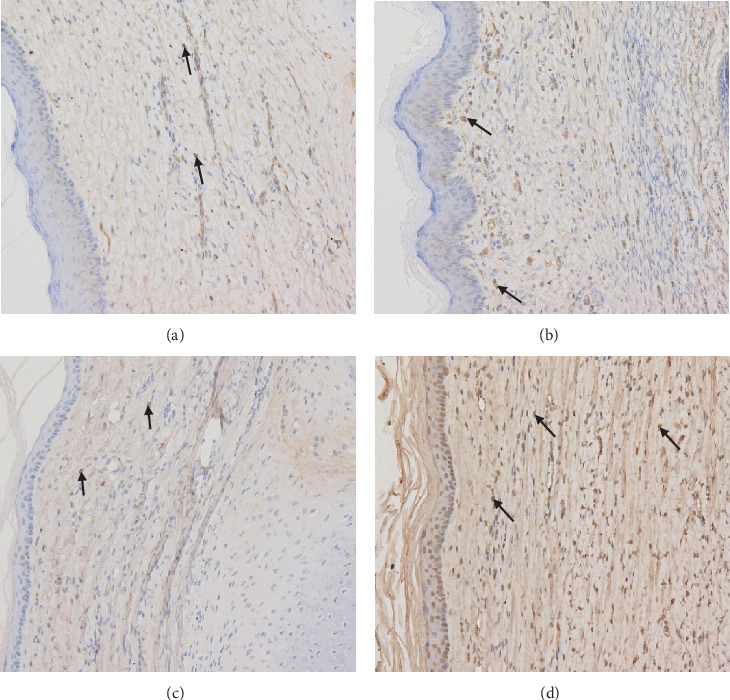
Expression of CD34 and VEGF (×200). (a, b) are positive expressions of CD34 in the treated group and the control group, respectively (as shown by the black arrow). (c, d) are positive expressions of VEGF in the treated group and the control group, respectively (as shown by the black arrow).

On days 30 and 45, the MVD value of the treated group was less than that of the control group, and the difference was statistically significant (*P* < 0.05); the MVD value of the treated group on day 45 was less than that on day 30 (*t* = 1.602, *P* = 0.127) (Table 2, Figures 3 and 4(a) and 4(b)).

### 3.4. VEGF

**Figure 5:**
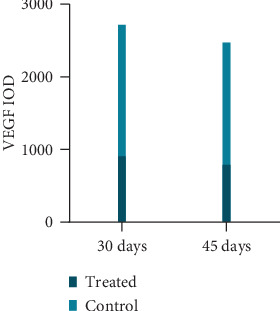
VEGF-positive IOD value of two groups at each time point.

On days 30 and 45, the VEGF expression of the treated group was less than that of the control group, and the difference was statistically significant (*P* < 0.05); the VEGF expression of the treated group on day 45 was less than that on day 30 (*t* = 1.379, *P* = 0.185) (Table 3, Figures 4(c) and 4(d) and 5).

### 3.5. Correlation Analysis

**Figure 6:**
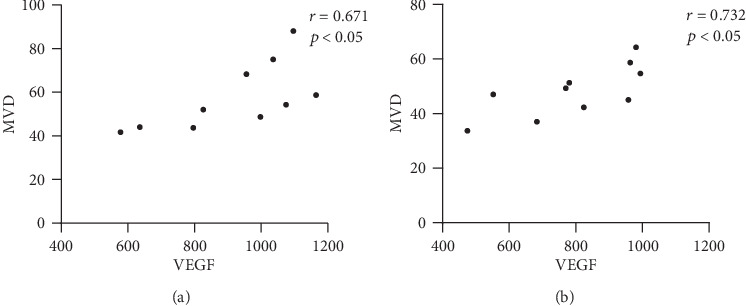
Correlation analysis of VEGF and MVD. (a) Correlation analysis of VEGF and MVD in the 30-day treated group. (b) Correlation analysis of VEGF and MVD in the 45-day treated group.

There was a positive correlation between MVD value and VEGF expression in the treated group on day 30 (*r* = 0.671, *P* < 0.05) and day 45 (*r* = 0.732, *P* < 0.05) (Figure 6).

## 4. Discussion

Although scarring is inevitable in regenerative repair and wound healing after tissue injury, HS incidence is extremely high, ranging from 40% to 91% for various reasons [12], and Asian people tend to form scars after skin injury [13]. It is widely believed that HS, a pathological scar, is formed due to blocked apoptosis and excessive generation of fibroblast, decreased degradation and increased synthesis of extracellular matrix collagen, and disturbed regulation of various cytokines [14].

In previous studies of BTXA injection in different periods, it was found that the effect of BTXA injection immediately after modeling was equivalent to that of the injection 10 days after modeling, with wound healing unaffected. Specifically, the latter was more effective in reducing the number of fibroblasts, while the former could significantly reduce the number of microvessels in the scar tissue according to HE staining. Thus, the study was aimed at further verifying the effects of BTXA on microvessels in HS tissues (unpublished data).

As angiogenesis is essential to wound healing, and multiple stages benefit from the participation of blood vessels and their bioactivators, angiogenesis and revascularization are critical for wound healing [15]. Angiogenesis can be quantified by MVD instead of direct measurement. CD34, a 110 kDa transmembrane glycoprotein of unknown function, is considered an important marker of angiogenesis and can represent MVD in tissues [16]. VEGF is the most potent angiogenic cell growth factor which is known to stimulate the migration of endothelial cells and increase vascular permeability, playing an extremely important role in angiogenesis and tissue repair [17, 18]. The aberrant hyperplasia of local microvessels is an important factor in HS formation, and VEGF and CD105 are highly expressed in pathological scar tissue, suggesting that abnormal vascular hyperplasia is involved in the development of pathological scars [18–20]. In addition, related studies have also confirmed that the degree of scar hyperplasia is related to microvessel flow [21].

According to the results of the study, the scar height, VEGF expression, and MVD value in scar tissue were all reduced in the BTXA-injected group, suggesting that the inhibition of scars by BTXA is related to the reduction of angiogenesis. By using models of scarless and fibrotic repair, Wilgus et al. [22] confirmed that fibrotic wounds had relatively more blood vessels with higher VEGF expression and that neutralizing VEGF could reduce vascularity and inhibit scar formation. VEGF may also regulate scar formation indirectly by stimulating endothelial cells or affecting inflammatory cells [23].

Arnold et al. [24] found that the VEGF-165 mRNA expression was decreased in the BTXA-injected group compared with the saline-injected group in a rat pedicled abdominal flap model study, while VEGF-165 mRNA was positively correlated with angiogenesis [25]. A significant correlation was found between CD34 and VEGF in an oral submucous fibrosis study by Sharma et al. [26], which was similar to the results herein. This study also revealed that the scar height, MVD value, and VEGF expression in the treated group on day 45 tended to be lower than those on day 30, which may be related to the decreased VEGF expression, vasoconstriction, occlusion, and chronic remodeling of scar tissue in the late stages of wound healing, while BTXA may be mainly effective in early healing.

At present, there are some studies on the effects of BTXA on VEGF and angiogenesis. Specifically, Eliane et al. [27] found that the distribution of proteoglycans and glycosaminoglycans, as well as VEGF expression, was reduced wherever BTXA was injected in the masseter muscles of transgenic mice. Similarly, Che et al. [28] established a rat model of supraspinatus tendon injury and injected BTXA into the supraspinatus muscle after modeling. The BTXA-injected group was compared with the saline group 4 weeks after injection, and VEGF expression in the BTXA-injected group was found to be significantly lower. Zhou et al. [29] established a rat model of prostatic hyperplasia and injected BTXA into the prostate tissue. BTXA was then observed to inhibit VEGF expression, thereby inhibiting angiogenesis in the prostate tissue and promoting prostate shrinkage, which was consistent with the results of this study.

However, the correlation of botulinum toxin versus VEGF and angiogenesis is still controversial. Some previous studies demonstrated conflicting results. Park and Park [30] demonstrated that BTXA treatment might increase the expression of VEGF and yield protection against ischemia-reperfusion injury depending on increasing angiogenesis. Other studies also concluded that the group with BTXA treatment had higher VEGF expression in a rat transplantation model or transverse rectus abdominis myocutaneous flap in a rat model [31, 32]. The concentration of BTXA used in these studies was 10 IU/mL, but Gugerell et al. [33] found that high BTXA concentration of 20 IU/mL might reduce VEGF expression and inhibit angiogenesis. In addition, Harper and Bates [34] highlighted that VEGF has a proangiogenic isoform and an antiangiogenic isoform, and the antiangiogenic VEGFxxxb isoforms might be switched to the proangiogenic VEGFxxx isoforms via splicing factors [35]. Thus, the VEGF isoform balance and the BTXA concentration might cause the conflicting result of correlation between BTXA and VEGF, which still need further research.

## 5. Conclusion

These results indicate that the injection of BTXA immediately after modeling can inhibit VEGF expression and reduce angiogenesis in tissues, thereby inhibiting HS formation in rabbit ears. However, more ample research is still needed to investigate the biology effects of BTXA on microvessels during the process of wound healing.

## Figures and Tables

**Table 1:** Scar height of two groups at each time point (mm).

	Day 30	Day 45
Treated	0.59 ± 0.07	0.56 ± 0.07
Control	1.12 ± 0.08	0.98 ± 0.07
*t*	-19.153	-16.171
*p*	0.000	0.000

**Table 2:** MVD value of two groups at each time point (/scope).

	Day 30	Day 45
Treated	57.43 ± 15.27	48.33 ± 9.46
Control	94.20 ± 19.45	78.50 ± 12.01
*t*	-4.703	-6.239
*p*	0.000	0.000

**Table 3:** VEGF-positive IOD value of two groups at each time point.

	Day 30	Day 45
Treated	916.20 ± 199.35	797.93 ± 183.99
Control	2724.39 ± 988.03	2480.58 ± 940.73
*t*	-5.673	-5.551
*p*	0.000	0.000

## Data Availability

The data used to support the findings of this study are available from the corresponding author upon request.
